# *In vivo* dosimetry and shielding disk alignment verification by EBT3 GAFCHROMIC film in breast IOERT treatment

**DOI:** 10.1120/jacmp.v16i1.5065

**Published:** 2014-01-08

**Authors:** Mara Severgnini, Mario de Denaro, Marina Bortul, Cristiana Vidali, Aulo Beorchia

**Affiliations:** ^1^ Department of Medical Physics A.O.U. Ospedali Riuniti Trieste Italy; ^2^ Department of Surgery A.O.U. Ospedali Riuniti Trieste Italy; ^3^ Department of Radiation Oncology A.O.U. Ospedali Riuniti Trieste Italy

**Keywords:** breast IOERT, *in vivo* dosimetry, radiochromic film EBT3, radiation shielding

## Abstract

Intraoperative electron radiation therapy (IOERT) cannot usually benefit, as conventional external radiotherapy, from software systems of treatment planning based on computed tomography and from common dose verify procedures. For this reason, *in vivo* film dosimetry (IVFD) proves to be an effective methodology to evaluate the actual radiation dose delivered to the target. A practical method for IVFD during breast IOERT was carried out to improve information on the dose actually delivered to the tumor target and on the alignment of the shielding disk with respect to the electron beam. Two EBT3 GAFCHROMIC films have been positioned on the two sides of the shielding disk in order to obtain the dose maps at the target and beyond the disk. Moreover the postprocessing analysis of the dose distribution measured on the films provides a quantitative estimate of the misalignment between the collimator and the disk. EBT3 radiochromic films have been demonstrated to be suitable dosimeters for IVD due to their linear dose‐optical density response in a narrow range around the prescribed dose, as well as their capability to be fixed to the shielding disk without giving any distortion in the dose distribution. Off‐line analysis of the radiochromic film allowed absolute dose measurements and this is indeed a very important verification of the correct exposure to the target organ, as well as an estimate of the dose to the healthy tissue underlying the shielding. These dose maps allow surgeons and radiation oncologists to take advantage of qualitative and quantitative feedback for setting more accurate treatment strategies and further optimized procedures. The proper alignment using elastic bands has improved the absolute dose accuracy and the collimator disk alignment by more than 50%.

PACS number: 87.55.kh

## I. INTRODUCTION

After surgically removing a malignant tumor, radiation therapy is usually recommended to prevent local disease recurrence from any residual cancer cells. Intraoperative radiation therapy (IORT) delivers concentrated and precise dose of radiation just after the surgical tumor removal (typically 10–25 Gray).^(1)^ Intraoperative electron beam radiation therapy (IOERT), that is based on treating with electron beams rather than X‐rays, has a homogenous dose distribution, limits penetration beyond the tumor bed, and delivers the required dose much more rapidly than X‐ray beams.

In order to properly treat the desired volume, it is necessary to take into account the surface dose, the depth dose curve, and the type of shield used to protect healthy organs and tissues. In particular, breast IOERT requires the protection of the tissues underneath the target volume, such as the heart and lungs. This is achieved by the surgeon positioning a shielding disk between the residual breast and the pectoralis fascia. The optimum size of the shielding disk depends upon the applicator chosen for the treatment.

Measurements of entrance dose during treatments are necessary to identify systematic and random errors and are recommended to guarantee a high quality of the treatment itself. *In vivo* dosimetry (IVD) is particularly needed in IORT because of the absence of software systems of treatment planning based on computed tomography.^2^, ^3^

As described in recent studies,^4^, ^5^ the setup of normal tissue protection and applicator placement are closely correlated with two very relevant risks in IOERT treatments: misalignment and wrong orientation of the shielding disk.

The particular position of the IOERT shielding disk is a very suitable location for performing film dosimetry. For this purpose, a new generation of radiochromic films, EBT3, has been chosen. This kind of radiochromic film has become an important tool to verify dose distributions in various fields, such as highly conformal radiation therapy and IMRT.^(6)^

A properly calibrated EBT3 radiochromic film can be fixed on both faces of the disk, allowing one to obtain two images providing a detailed bidimensional dose distribution. From the first image, obtained just below the target organ, it is possible to measure the absolute entrance dose in that position. Moreover, the visual analysis of the image can provide the surgeon with an effective feedback about the actual homogeneity obtained in the reconstruction of the target. From the second image positioned beyond the shielding disk, it is possible to evaluate the fraction of the dose passing through the disk and arriving at the underlying healthy tissue. There are several types of attenuation disks, made of several combinations of materials, and particular attention has to be paid in choosing the best one to achieve the desired attenuation, avoiding excessive backscattered radiation.^(7)^

This procedure for performing *in vivo* dosimetry has been carried out on 37 patients treated with IOERT. The data obtained from the radiochromic dosimeters were processed by customized software and the dose parameters were collected into a database of patients, which proved to be very useful in improving the treatment quality.

## II. MATERIALS AND METHODS

All treatments were performed by an IOERT‐dedicated electron beam accelerator, the MOBETRON (IntraOp Medical, Inc. Santa Clara, CA).

Selected patients have been submitted to breast IOERT, as preliminary boost with prescribed dose of 10 Gy, to be followed later by conventional external fractioned whole‐breast radiotherapy (50 Gy in 25 fractions).

**Figure 1:**
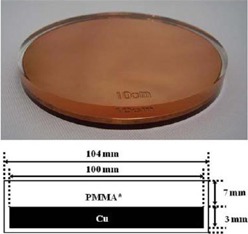
Sketch of the attenuator disk, showing the stack of PMMA and Cu layers.

In 90% of patients, we employed 8 cm diameter shielding disks provided by IntraOp, made by stacking a 5 mm polymethyl methacrylate (PMMA) layer, a 3 mm copper layer, and 2 mm PMMA layer, as shown in Fig. 1.

We measured the transmission and backscatter of the disk at the buildup depth with GAFCHROMIC film (EBT3) (International Specialty Products, Wayne, NJ) in water‐equivalent solid phantom (RW3; PTWFreiburg, Germany) by using a Varian Clinac (Varian Medical Systems, Palo Alto, CA) 2100C at 6 and 9 MeV. The obtained results are very similar to those described in literature.^8^, ^9^

The use of shielding disk also during IOERT boost delivery allowed better target reconstruction, and easier collimator setup and dose optimization.

### A. Data acquisition

**Figure 2:**
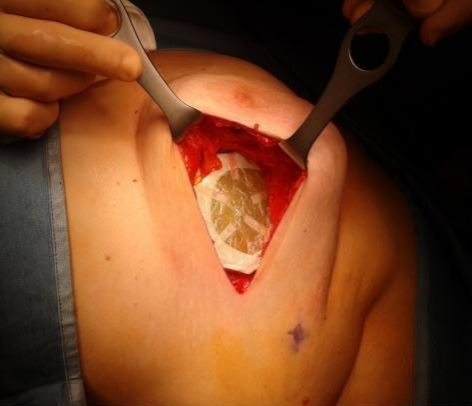
Disk placement *in vivo* behind a patient's breast parenchyma before the IOERT. The GAFCHROMIC film positioned on the top of the disk is visible.

Before the treatment, two layers of radiochromic film, GAFCHROMIC EBT3, cut to the same size of the shielding disk, were wrapped in a layer of protecting plastic film, then fixed by sterile tape to both sides of the shielding disk. Then the surgeon placed it beneath the breast tissue of the patient during the treatment preparation, as shown in Fig. 2.

**Figure 3:**
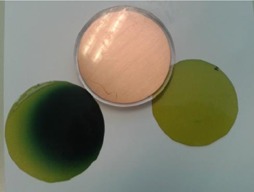
Postirradiation GAFCHROMIC EBT3 films previously positioned on the two sides of the attenuator disk.

Figure 3 shows the attenuator disk and the two GAFCHROMIC films that were positioned on the two sides during the irradiation.

The dark spot on the left film (top film) corresponds to the radiation field and — in this particular case— the film and consequently the shielding disk were not perfectly aligned.

On the other hand, the film on the right, positioned on the bottom side, is irradiated with a negligible dose and preserves the original colour.

From the image related to the film positioned on the top of the disk, we estimated the average absolute dose in a central area (2 cm diameter) of the exposed field to obtain the percentage difference from the expected dose.

From the image related to the lower position film we were able to measure the dose that actually crosses the shielding disk, which should be negligible.

We measured the backscatter for the two possible orientation of the disk obtaining a negligible value when the larger layer of PMMA is on the top, and a value of about 10% in the other orientation due to electrons backscatter produced by the copper layer. In order to identify the best disk orientation (larger layer of PMMA on the top), an orientation mark on the shielding disk is written with surgical marker before placement.

Geometrical analysis of the radiation field detectable on the top film has been performed so as to have information on the misalignment between the treatment field and the protective disk, and to evaluate the amount of dose that escapes from the shielding towards the healthy tissues.

After the treatment, the radiochromic films were recovered, and the plastic envelopment removed in order to work with a perfectly clean surface. The cleaned films were read by a CCD scanner EPSON Expression 10000XL (US Epson, Long Beach, CA) and the obtained images were processed and analyzed by a customized home‐made software.

### B. GAFCHROMIC calibration

Each batch of GAFCHROMIC film was calibrated in dose with MOBETRON to provide a bidimensional distribution in term of absolute dose. Following EBT3 GAFCHROMIC handling guide,^(10)^ the films were marked to keep track of the film orientation, cut in pieces of $5\times 5\;{\text{cm}}^{2}$, handled with gloves, and stored in order to minimize exposure to light.

While the past radiochromic films are sensitive to temperature variations, EBT2‐EBT3 GAFCHROMIC film has been demonstrated to be temperature independent, as shown by Andres et al.^(11)^

Moreover, since high doses are involved, we wait at least 24 hrs to read and process GAFCHROMIC films, both in the calibration procedure and *in vivo* dosimetry, in order to give time for the stabilization of the developing process, in agreement with recent study results.^(6)^

The GAFCHROMIC EBT3 calibration was done in solid water‐equivalent phantom (RW3‐PTW), for the two energies usually involved in breast treatments (6 and 9 MeV) and with the reference collimator (10 cm diameter) that was placed perfectly adherent on the surface of the slab phantom. We measured absolute dose at the calibration reference depth (IAEA 398 Code of Practice for Dosimetry) with an ionization chamber (Advanced Markus PTW) and we placed the GAFCHROMIC films in the same position. Then each $5\times 5\;{\text{cm}}^{2}$ film was irradiated at increased dose steps. After 24 hrs, we read all the irradiated films with a flatbed scanner (EPSON 10000XL). Films are positioned in the central area of the scanner (i.e., $12\times 12\;{\text{cm}}^{2}$) where it has a uniform response and there is no need for spatial dependent nonuniformity correction.^(12)^

The calibration curve for each energy was obtained as a function of the darkness reading of the film with respect to the dose increase. EPSON Expression 10000 XL scanner generates a digital image in 48 bit RGB format that is postprocessed by considering only the green channel (G) that is the most sensitive for such a high dose. Then the net optical density has been calculated by using the Devic algorithm.^13^, ^14^

**Figure 4:**
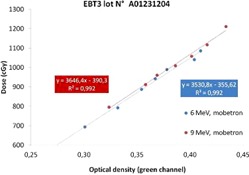
EBT3 calibration curve measured in solid phantom; in the dose range of interest there is a linear relationship between the dose and the optical density.

Figure 4 shows an example of calibration curve in the dose range of interest.

**Figure 5:**
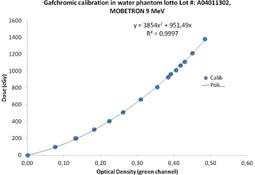
EBT3 calibration curve in water phantom; the dose versus optical density presents a quadratic behavior.

One batch has been also calibrated in PTW MP3‐XS water tank, obtaining results very similar to the calibration in solid water phantom, with a discrepancy lower than 1%. In the water tank calibration, we measured the dose at the reference depth with the same ionization chamber and then we put the films at the same depth on the ion chamber support protected by very thin plastic envelopes. The results for calibration in water tank for the 9 MeV electron beam are plotted in Fig. 5. It is remarkable to observe that, in this wider range (0–14 Gy), the dose behavior versus the optical density is not linear anymore, but it presents a quadratic increase.

### C. Image processing

Processing of films images has been standardized by using an in‐house built software based on ImageJ.^(15)^

The main steps of the analysis are:

1. Load the collimator and shielding disk size.
2. Select the green channel and converting pixel values into optical density.
3. Convert optical density into absolute dose by means of the calibration curve.
4. Enhance the contrast visibility by using a 16 colors look‐up table.
5. Overlap graphically the shielding disk circumference and semi‐automatic drawing of the circle corresponding to the beam size (dependent on the applicator diameter).

**Figure 6:**
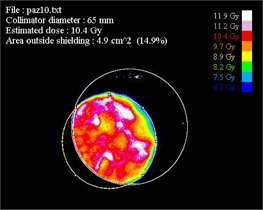
Output image provided by the software analyzing of the GAFCHROMIC film positioned on the top of the disk for Patient #10. Isodose areas are represented with the same colors listed on the right.

The software finally provides the mean absolute dose in the upper side of the shielding disk, the percentage of the beam escaping from the shielding disk due to misalignments, and the final image frame with superimposition of the main evaluated parameters (see Fig. 6).

## III. RESULTS & DISCUSSION

Thirty‐seven patients were treated with mobile electron beam accelerator, the Intraop Mobetron. Every morning before treatments a quality control with a dedicated phantom provided by the company was done for each energy, as described by Mills et al.,^(16)^ in order to verify the constancy of dose and energy, as suggested by AAPM Task Group 72 recommendation.^(17)^

The average variation over two years of treatment of the output values and energy relative to the reference condition is less than 1%, with a maximum value of 2%. The maximum energy change produced an equivalent shift of less than 1 mm on the depth‐dose curve. This is in agreement with past publication results obtained with the same mobile accelerator.^(18)^

Table 1 reports the results on the treated patients, showing the percentage difference between measured and prescribed dose and the area of the field that escapes from the shielding.

In 95% of the cases, good agreement with the prescribed dose has been obtained, with an average difference less than 4%. The lower position film measures the dose under the copper PMMA shield that is always less than 40 cGy.

In two patients (∼5%) we observed an absolute dose 30% lower than the predicted and this might be due mainly to clinical mishandling such as wrong thickness determination, blood losses increasing the effective thickness, and no perfect matching between collimator and target.

**Table 1:** Results for the 37 patients treated in this study. In the first six patients, the dimensions of GAFCHROMIC film were smaller than the disk's and it is not possible to estimate the area of the radiation field that escapes outside the shield; the data is not available (n.a.)

*N.Rt*	*Energy MeV*	*Collimator Diameter cm*	*Collimator Edge Aperture Angle °*	*Difference Expected Dose vs. Measured Dose %*	*Area Outside Shielding cm^2^*
1	9	6.5	0	−2	n.a.
2	9	7	15	−2	n.a.
3	9	5.5	0	−3	n.a.
4	9	5.5	0	−5	n.a.
5	6	5.5	15	−1	n.a.
6	9	6	15	−10	n.a.
7	9	5.5	0	5	1.2
8	9	6	0	−2	8.2
9	6	6	0	−39	1
10	9	6.5	30	4	4.9
11	9	7	30	4	21.2
12	6	6.5	0	−1	8.7
13	9	6	0	−7	5.5
14	9	5.5	15	5	2.1
15	9	5.5	15	−35	13.3
16	9	6	15	1	5.8
17	9	6	15	3	0
18	9	5	15	8	0
19	9	5	15	3	11.7
20	9	5	15	0	0.3
21	6	6	15	−1	2.6
22	9	5	15	−1	0
23	9	5.5	0	4	4.3
24	9	5	0	−9	3
25	9	5.5	0	3	0
26	9	5.5	15	1	8.7
27	9	5.5	15	3	4.8
28	9	5	30	7	1.4
29	6	5	30	−9	0
30	9	6	15	−8	0
31	9	5.5	30	−3	8.4
32	6	5.5	15	1	6.8
33	9	5.5	15	−4	4.1
34	6	5.5	15	−1	0
35	9	6.5	0	−5	11.4
36	9	5.5	15	−5	1.5
37	9	5.5	30	−1	4.7

However the results were very useful to the surgeon, radiation oncologist, and medical physicist to improve the radiotherapy procedures. After the treatment, the uniformity of the exposed area was visually evaluated by the surgeon to obtain an effective feedback on the actual homogeneity of the reconstructed target. Moreover, the percentage of the entrance beam that escapes from the protecting disk gave important information about the unavoidable misalignment between the exposure field and the protective disk.

**Figure 7:**
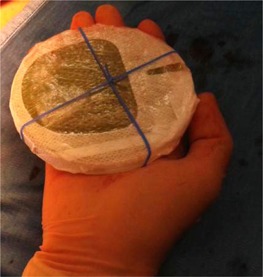
New shielding setup with the surgical elastic band.

To avoid unknown peripheral target thickness, the surgeon has increased the sampling of the target thickness, and to avoid air gap the radiation oncologist checks the perfect matching between collimator and target. Furthermore, to avoid shielding disk misalignment, the surgeon and the radiation oncologist have decided to design a new shielding setup, implementing an elastic band fixed by stitches to the pectoralis fascia, thus preventing the slippage of the disk (see Fig. 7).

The optimized setup described above has been implemented, starting from Patient #16, and it has improved the treatments, as shown in Table 1 — dose measurements are more in agreement with the expected dose and the area outside the shielding is relevantly reduced.

**Figure 8:**
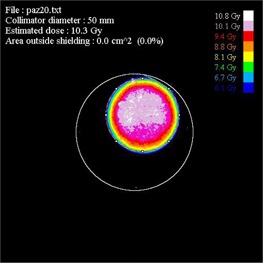
Example of excellent collimator disk alignment in Patient #20; 0 cm^2^ of area outside the shielding.

The last 21 cases treated with the described improved procedure show particularly good results in terms of dose delivered, homogeneity, and shielding alignment. Compared to the first 16 patients, those treated with our best practice showed an improvement of more than 50% in terms of absolute dose and disk alignment. In fact, the field area outside the shield is reduced from an average value of 7.3 cm^2^ to 3.6 cm^2^. A perfect collimator disk alignment (no field outside the disk) has been obtained in eight patients (see Fig. 8).

## IV. CONCLUSIONS

The IOERT providing high‐dose treatment requires high accuracy in determining the dose delivered to the target. For this reason *in vivo* measurement represents a very important test to evaluate the actual dose delivered. This paper has shown that GAFCHROMIC EBT3 films positioned on the shielding disk are effective dosimeters for the IOERT without any drawbacks in the clinical practice. From the postprocessing of the irradiated EBT3 films it is possible to obtain a detailed dose map of the target, the absolute dose delivered, and an estimate of the area of the field outside the shielding. Furthermore, the dose map provides surgeons with important information about the homogeneity of the reconstructed target.

This method has resulted in a multidisciplinary discussion between physicists, radiation oncologists, and surgeons, leading the staff to undertake a new procedure to reach the best practice.

With the new approach we were able to obtain very good results in terms of dose delivered, homogeneity, and shielding alignment. We have obtained a 50% reduction of the dose area outside the shielding disk and of the average difference between actual dose and expected dose.
